# Potential of Sub-GHz Wireless for Future IoT Wearables and Design of Compact 915 MHz Antenna

**DOI:** 10.3390/s18010022

**Published:** 2017-12-22

**Authors:** Adolfo Di Serio, John Buckley, John Barton, Robert Newberry, Matthew Rodencal, Gary Dunlop, Brendan O’Flynn

**Affiliations:** 1 Tyndall National Institute, University College Cork, Dyke Parade, T12R5CP Cork, Ireland; adolfo.diserio@tyndall.ie (A.D.S.); john.barton@tyndall.ie (J.B.); brendan.oflynn@tyndall.ie (B.O.); 2 Sanmina Corporation, 13000 S. Memorial Parkway, Huntsville, AL 35803, USA; robert.newberry@sanmina.com (R.N.); matthew.rodencal@sanmina.com (M.R.); 3 Sanmina Ireland, Rathealy Road, Fermoy, P61FX24 County Cork, Ireland; gary.dunlop@sanmina.com

**Keywords:** IoT, ISM band, small antenna, wearable, medical, SpO2, wireless sensor, wrist-worn antenna

## Abstract

Internet of Things (IoT) technology is rapidly emerging in medical applications as it offers the possibility of lower-cost personalized healthcare monitoring. At the present time, the 2.45 GHz band is in widespread use for these applications but in this paper, the authors investigate the potential of the 915 MHz ISM band in implementing future, wearable IoT devices. The target sensor is a wrist-worn wireless heart rate and arterial oxygen saturation (SpO2) monitor with the goal of providing efficient wireless functionality and long battery lifetime using a commercial Sub-GHz low-power radio transceiver. A detailed analysis of current consumption for various wireless protocols is also presented and analyzed. A novel 915 MHz antenna design of compact size is reported that has good resilience to detuning by the human body. The antenna also incorporates a matching network to meet the challenging bandwidth requirements and is fabricated using standard, low-cost FR-4 material. Full-Wave EM simulations are presented for the antenna placed in both free-space and on-body cases. A prototype antenna is demonstrated and has dimensions of 44 mm × 28 mm × 1.6 mm. The measured results at 915 MHz show a 10 dB return loss bandwidth of 55 MHz, a peak realized gain of −2.37 dBi in free-space and −6.1 dBi on-body. The paper concludes by highlighting the potential benefits of 915 MHz operation for future IoT devices.

## 1. Introduction

The continuous growth of the number of wireless sensor-actuator nodes has led to the creation of the Internet of Things (IoT) [1]. Among them, wearable devices represent a significant proportion. In fact, according to the forecasts, in 2021 there will be 928 million connected wearable units worldwide [2], and the revenue worldwide in the same year is expected to be valued at 95,270.0 million USD [3]. Specifically, medical wearables represent a rapidly growing market [4]. At the present time, a large number of commercial wearable health and fitness monitors incorporate heart rate monitoring (HRM) [5,6,7,8,9]. The addition of SpO2 can provide new functionality to predict activities such as human kinetic and sleep patterns for medical and sports applications. The main principle behind an optical SpO2 sensor uses a photoplethysmography (PPG) technique to detect blood volume changes in the micro-vascular bed of tissue. Pulse oximetry is a noninvasive method for accurately estimating blood oxygen saturation (SpO2) in the human body. 

In terms of realizing wearable sensors, wireless communications capability is a key enabling technology. This is especially true in IoT applications as it enables information exchange with the cloud for data collection, analysis and provision of near real-time responses [10] and addressing the future increasing demands of IoT-based health-care services [11]. 

**Figure 1:**
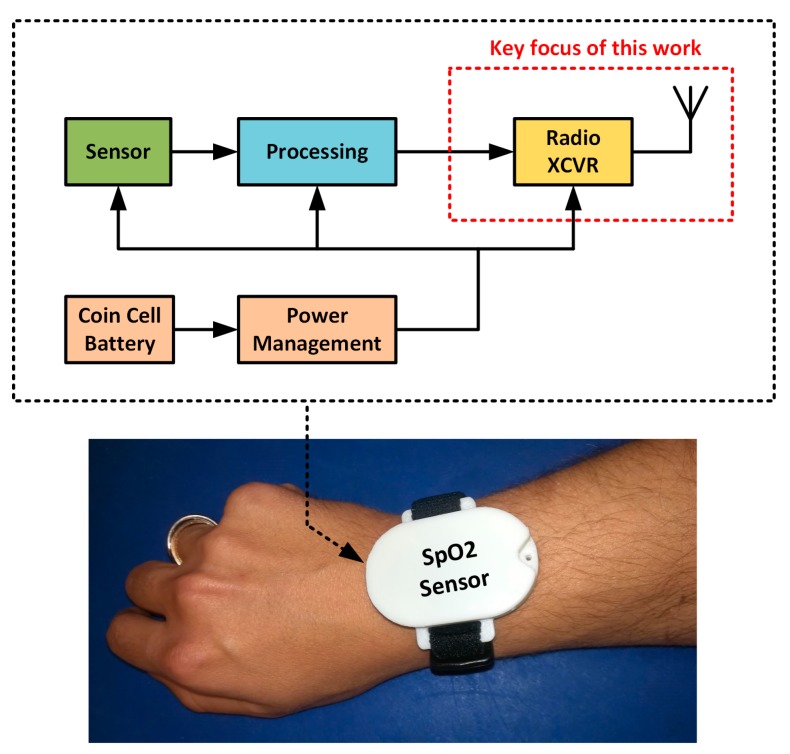
Wrist-worn wireless SpO2 sensor in final configuration with corresponding system block diagram.

In this paper, the design of a wrist-worn wireless SpO2 sensor is proposed. Figure 1 presents a system block diagram composed of an SpO2 sensor, a processing block, a radio transceiver (XCVR) and an antenna as well as a battery and power management block. The key focus of this work concerns the radio XCVR and antenna. A key challenge concerns the antenna, which is constrained in size by the dimensions of the overall system, and which can accommodate a board of dimensions approximately 44 mm × 28 mm. 

At the present time, the 2.45 GHz ISM band [12] is invariably used for the provision of IoT wireless connectivity for medical wearable applications as it offers advantages such as small antenna size and availability of a large number of wireless standards e.g. Bluetooth [13], Zigbee [14] and Wi-Fi [15]. Other wireless bands for medical applications include the Medical Device Radiocommunications Service (MedRadio) band [16] and 915 MHz ISM band [12] in United States, the 433 MHz ISM band and the 869.5 MHz Short Range Device band in Europe [17]. These frequency bands can offer potential performance advantages in terms of reduced path loss, band congestion and channel stability that we investigate in this work. 

In the literature, several works have been published that proposes integrated antenna designs for wrist-worn devices such as smart watches, where a large number are proposed for a working frequency of 2.45 GHz, e.g., [18,19,20]. In particular, in these works, the antenna dimensions are comparable to the dimensions available for the radio XCVR plus antenna board in the wrist-worn wireless SpO2 sensor proposed. Several examples of antenna designs for wrist-worn devices operating also in Sub-GHz frequencies are reported in [21]. These designs use watch Printed Circuit Board (PCB) of dimensions 40 × 40 mm^2^ with a metal belt of dimensions ~ 100 × 20 mm^2^ as an integral part of the antenna.

The interaction between human body and antenna is a key issue with wearable wireless devices. In fact, the human body is characterized by a specific conductivity and by several layers of material with different thicknesses and different dielectric permittivity. These parameters are also frequency dependent and when the antenna is placed in proximity of the human body it can experience a variation of the impedance characteristics, namely frequency detuning, with respect to a free-space scenario [22]. From this point of view, Sub-GHz frequencies have a strong potential for wearable medical applications, as using these frequencies, the interaction between human body and antenna decreases with less deterioration in antenna and wireless communications performance [23].

Power consumption represents another critical factor for the design of battery powered medical wearable devices. In particular, the availability of ultra-low power radio transceivers allows the extension of the battery life-time and hence the reduction of battery recharging or replacement operations.

In this work, the aim of the authors is to investigate the use of a Sub-GHz wireless frequencies as an alternative to 2.45 GHz. In Section 2, the potential of the 915 MHz and 2.45 GHz ISM bands are compared focusing on wireless link characteristics, device form factor and power consumption. This analysis shows the potential benefits of using the 915 MHz ISM band but also outlines a number of antenna design challenges that need to be addressed and overcome. In Section 3, the antenna design process is discussed in detail, with a particular focus on the 3D electromagnetic modeling of the structure and on the antenna topology. In Section 4, the results of full-wave electromagnetic simulations are presented in detail. In Section 5, a prototype antenna is demonstrated. The measured results show that the antenna exhibits desirable bandwidth characteristics as well as a robustness to detuning. Finally, in Section 6, the potential benefits of 915 MHz operation for future IoT devices and in particular for wrist-worn devices is highlighted and is followed by a summary of the main conclusions of this work in Section 7.

## 2. Comparison of Wireless Performance in the 915 MHz and 2.45 GHz ISM Bands 

In this section, an analysis of potential wireless link performance for a wrist-worn wireless SpO2 sensor device is investigated by considering two frequency bands, namely the 915 MHz and 2.45 GHz ISM Bands. In order to compare potential performance in both the frequency bands under examination, several factors need to be investigated. These factors can be divided into two main groups: wireless link characteristics and DC power consumption. The wireless link characteristics comprise free-space path loss (FSPL), and antenna gain constraints due to device form factor. DC power consumption is related to the wireless protocol and to the RF Integrated Circuit transceivers that implements the specific protocol as well as the wireless channel coexistence with other systems using different wireless standards [24]. 

### 2.1. Wireless Link Characteristics

**Figure 2:**
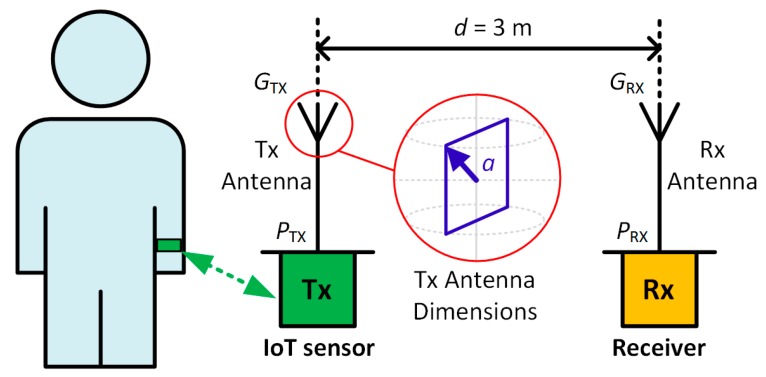
IoT sensor (Tx) placed on the human wrist communicating with a remote receiver device (Rx).

The application proposed in this work concerns IoT smart wearable sensor devices used for medical monitoring applications. This class of applications is referred to as Body Area Networks (BANs). Different possible wireless communication scenarios have been identified from the IEEE 802.15 WPAN Task Group 6 (TG6) Body Area Networks [25], such as the radio channel between implanted devices, implanted device to body surface, implanted device to external, body surface to body surface and body surface to external radio channel [26]. The latter represents the scenario of interest in the proposed system and it is depicted in Figure 2 where the transmitting device Tx represents the wrist-worn wireless SpO2 sensor. Tx communicates with a remote receiver Rx, placed at a given distance *d* by means of Tx Antenna and Rx Antenna. Rx is also connected to the Internet. The transmitter and receiver antenna gains are denoted $G_{TX}$ and $G_{RX}$, the transmitted and received power are denoted $P_{TX}$ and $P_{RX}$.

Initially, the free-space path-loss is evaluated considering the scenario depicted in Figure 2 for the two frequency bands under investigation. To this aim, referring to Figure 2, it has been assumed that the communication between two antennas Tx and Rx take place in line-of-sight and in free-space scenarios, moreover the distance between Tx and Rx antenna is *d* = 3 m. The FSPL have been calculated using the following equation [27,28],

(1) $$\mathrm{FSPL}(\mathrm{dB})\;=\;10\;\log_{10}{\left(\frac{4\pi d}{\lambda_{0}}\right)}^{2}\;.$$

From (1), the FSPL when the system operates at a frequency of 915 MHz, ${\mathrm{FSPL}}_{915\mathrm{MHz}}$ is 41.2 dB, and for an operating frequency of 2.45 GHz, ${\mathrm{FSPL}}_{2.45\mathrm{GHz}}$ is 49.8 dB and $\lambda_{0}$ is the free-space wavelength. Therefore, under the same conditions the FSPL for an operating frequency of 2.45 GHz is 8.6 dB greater than the case of an operative frequency of 915 MHz. Hence, in the first case the transmitted signals experience a more severe attenuation than the latter.

With respect to the form factor, in the proposed application the transmitting antenna needs to be integrated in a wrist-worn wireless SpO2 sensor device of finite dimensions. However, in [29] the fundamental performance limits of antennas with respect to electrical size have been investigated. This work relates the maximum theoretical gain achievable from an antenna given to its physical size and its working frequency. Referring to Figure 2, the maximum transmitting antenna dimension is 2*a*, where *a* represents the minimum radius of a sphere enclosing the antenna element [29]. In this specific case, dimension *a* is limited to 22 mm as this is a physical constraint of the wrist-worn wireless SpO2 sensor depicted in Figure 1. It follows an analysis of the impact of the form factor constraints on the antenna performance for the transmitting antenna gain *G*_TX_ using [29]

(2) $$G_{TX}\;=\;{\left(\beta a\right)}^{2}\;+\;2({\beta a),\;\mathrm{where}\;\beta \;=\;2\pi /\lambda }_{0}.$$

In particular, the maximum theoretical gain using (2) for the Tx antenna at 915 MHz is $G_{TX\_915MHz}\;=\;+0.09\;\mathrm{dBi}$ while the maximum theoretical gain at 2.45 GHz is $G_{TX\_2.45GHz}=\;+5.48\;\mathrm{dBi}$. These calculations show that theoretically, given the form factor constraints above, the maximum gain achievable by an antenna resonant at 915 MHz is around 5.4 dBi lower than the maximum theoretical gain of an antenna resonant at 2.45 GHz.

Considering the Tx antenna gains $G_{TX\_915MHz}$ and $G_{TX\_2.45GHz}\;$ and the FSPL calculated above from (1) for both the frequencies under test it is possible to calculate the received power from Rx antenna using the Friis equation [30] reported below. An important assumption in the following analysis is that the gain of the receiver antenna $G_{RX}$ is specified as 0 dBi at both 915 MHz and 2.45 GHz as this represents the gain of an antenna with omnidirectional radiation characteristics that is typically used in a receiver hub. However, if more directivity is required, the value of $G_{RX}$ can be increased depending on the application. It has been also assumed that the transmitted power $P_{TX}\;$is 0 dBm.

(3) $$P_{RX}\left(\mathrm{dB}\right)\;=\;10\;\log_{10}\left(P_{TX}G_{TX}G_{RX}{\left(\frac{\lambda_{0}}{4\pi d}\right)}^{2}\right)$$

From this calculation, the power received when the system operates at a frequency of 915 MHz, $P_{RX\_915MHz}$ is equal to −41.1 dBm, when the system instead operates at 2.45 GHz the received power $P_{RX\_2.45MHz}\;$ is −44.3 dBm. In light of these results, it can be concluded that also considering a limitation on the antenna form factor, an improved link budget performance can be obtained operating in the 915 MHz ISM frequency band. 

Table 1 summarizes the calculated results for the scenario depicted in Figure 2. The parameters and units are displayed in the first two columns. The calculated results are summarized in the third and fourth columns with the calculated term differences between 915 MHz and 2.45 GHz ISM band results denoted ∆ (dB) displayed in the right-most column. 

In conclusion, a key advantage of 915 MHz operation is the greatly reduced free-space path-loss (FSPL), which is 8.6 dB lower than the case at 2.45 GHz. Taking into account also the limitation on the form factor of the wrist-worn wireless SpO2 sensor, even though the theoretical achievable antenna gain $G_{TX}$ is limited to approximately +0.1 dBi at 915 MHz when compared to +5.5 dBi at 2.45 GHz, there is a net increase in received power of approximately 3.2 dB assuming that *G*_RX_ is limited to 0 dBi. However, if it is possible to increase the value of *G*_RX_ by increasing the directivity of the receiving antenna or by employing additional Rx antennas, then the value of *G*_RX_ could be doubled (3 dB) by employing two receive antennas for example. In this case, the calculated results in Table 1 show that the received power at 915 MHz and 2.45 GHz is approximately the same or a 0.16 dB difference. 

### 2.2. DC Power Consumption 

As for the power consumption aspect, a contextualization regarding requirements of the proposed application in terms of data rate, maximum communication range, and maximum transmitted power allowed is required, in order to determine an optimal wireless protocol to use for the application as well as analyzing the respective radio transceiver power consumption. In particular, it can be assumed that the communication of sensed parameter in the proposed system requires a desired Data Rate of 10 kbps as SpO2 measurement does not require a large data payload [11]. In addition, a communication range of 3–6 m is assumed in order to allow freedom of movement to the user during wireless communications. The maximum transmitted power allowed for a wearable application is specified as 0 dBm as outlined in [31]. In this analysis, the power consumption will be considered in terms of current consumption in sleep mode, transmitting mode and receiving mode.

#### 2.2.1. Wireless Protocols 

In Table 2, a survey of potential Wireless Protocols for future IoT applications is reported. The overall protocols are characterized by different operating frequencies, maximum data rates, maximum communication ranges and network topologies and hence are optimized for specific applications. 

Bluetooth Basic Rate/Enhanced Data Rate (BR/EDR) protocol provides a maximum data rate of 3 Mbps, it can communicate only in peer-to-peer (P2P) mode and its target application is audio streaming [13]. Bluetooth Low Energy (BLE) also provides a high maximum data rate of 2 Mbps and is optimized to connect several devices such as fitness trackers, health monitors, PC peripherals and accessories. Moreover, it can enable several network topologies, e.g., P2P, Star and Mesh [41]. ZigBee is a protocol that provides a maximum data rate at 2.45 GHz of 250 kbps [14] and the possibility to implement P2P, Star and Mesh topologies. It is designed to provide interoperability for a range of applications such as home automation, health monitoring and telecommunication [14]. Z-Star is a proprietary protocol optimized for ultra-low power wireless sensor network applications; it provides a maximum data rate of 200 kbps, and the possibility to implement several kinds of network topologies including peer-to-peer (P2P), Star, Mesh and others. WiFi is a technology based on IEEE 802.11 standards that provides a maximum data rate of 54 Mbps at 2.45 GHz [34]. This protocol implements a Star network topology and is designed to allow connectivity for smart home products, localization applications, industrial sensor systems etc. [42]. HaLow is a low-power WiFi solution operating in the Sub-GHz frequency bands and provides a maximum data rate of 8.7 Mbps (under specific conditions, namely using a 2 MHz channel and 1 spatial stream) and a Star network topology. It is designed for building automation, healthcare, industrial, agricultural and other applications [35,43]. ANT/ANT+ are low data rate (maximum 60 kbps) and Ultra-Low Power wireless protocols suited for Wireless Sensor Network applications. It allows the implementation of P2P, Star and Mesh network topology. LoRa is a protocol for Low Power Wide Area Network for the communication of wireless battery powered nodes at a maximum data rate of 300 kbps [37,38]. UHF RFID is a protocol that allows deployment of wireless RFID tags to identify objects, with possibility to get further sensed information at a maximum data rate of 640 kbps [39,40].

As for the operating frequency, it can be noticed that the majority of the protocols are designed to work in the 2.45 GHz ISM Bandwidth, namely Bluetooth BR/EDR and BLE, ZigBee, WiFi and ANT/ANT+, among them ZigBee is designed also for operation in Sub-GHz band and WiFi can operate also in the 5.8 GHz ISM Band. The protocols that are designed specifically to operate at Sub-GHz ISM Band are then Z-Star, HaLow, UHF RFID and LoRa. 

As for the maximum communication range, Bluetooth protocols ANT/ANT+ and UHF RFID are short range protocols, which ensure a communication respectively up to 10 m, 5 m and 15 m. WiFi communication can operate over a maximum range of 30 m. On the other hand, Z-Star, ZigBee and HaLow protocols can cover a distance up to 100 m, while LoRa can cover a distance of 2 to 5 km in an urban area. 

Finally, focusing the attention on coexistence issues with external interfering network(s), most of the WBAN systems work in the 2.45 GHz ISM Band, but it represents an already crowded band because the pervasive presence of devices that operate at the same frequencies using different standards, such as Wi-Fi, Bluetooth, ZigBee and others [24,44]. This aspect suggests to follow a path toward the use of different radio protocols working at different frequencies in order to reduce the interference as well as to reduce multiple retransmissions and hence the power consumption and to improve the Quality of Service (QoS).

#### 2.2.2. Commercial RF Integrated Circuit Radio Transceivers

In Table 3, a survey of low-power commercially available Radio Frequency Integrated Circuit (RFIC) transceivers that implement the wireless protocols listed above is reported. The survey does not include an RFIC for HaLow to the author’s knowledge, shared information regarding RFIC characteristics is not available at the present time. The comparison and the choice of the RFICs are led by the assumptions related to the requirements for the communication of SpO2 sensed parameters in the application of interest made in Section 2.2. Namely, a Data Rate of 10 kbps is assumed and the maximum radio transmit power of 0 dBm is also assumed. For these reasons, RFIC devices that satisfy these conditions are chosen for comparison. The numeric values in Table 3 are now used for subsequent calculations of current consumption for each of the wireless protocols and the corresponding RFIC devices. 

In order to estimate the DC power consumption of each transceiver, the duty cycle associated with specific applications needs to be considered. Specifically, the duty cycle is defined as the ratio of the percentage of “transceiver ON time” over a specified period of time [51]. Hence, the duty cycle is affected by many factors, among them the size of the data payload to be transmitted, the wireless protocol used, and hence by the data rate [51,52], the number of nodes [53,54], network topology [55] and link quality [51].

The duty cycle δ is defined by the following equation [51]:

(4) $$\delta \;=\;\frac{T_{ON}{\;+\;T}_{TX}{\;+\;T}_{RX}{\;+\;(T}_{\mathrm{sync}}{/N}_{R})}{T_{\mathrm{frame}}}\;(1\;+\;PER)\;,$$

where $T_{ON}$ is the transmitter startup time, $T_{TX}$ is the time required to transmit data and overhead bits such as preamble, address and other necessary information. $T_{RX}\;$is the time required to receive the acknowledgement packet comprising of the overhead bits, $T_{\mathrm{sync}}$ is the time required for the transmission of synchronization information, $N_{R}$ is the number of cycles between two synchronization packets, $T_{\mathrm{frame}}$ is the time interval between the transmission of two data packets. For example in the following analysis, a sample rate of 1 sample per second corresponds to a frame rate of $T_{\mathrm{frame}}\;=$ 1 s. The quantity PER is defined as the Packet Error Rate. In particular, the term (1 + PER)  takes into account the effect of the link quality and indicates how the packet retransmission can affect the duty cycle and hence the power consumption. The calculation of δ for each protocol assumes that $T_{ON}\;=$ 0 and a PER of 1% has been assumed. Equation (1) can be now rewritten as

(5) $$\delta \;=\;\frac{{\;T}_{TX}{\;+\;T}_{RX}\;}{T_{\mathrm{frame}}}\;(1\;+\;PER)\;.$$

**Figure 3:**
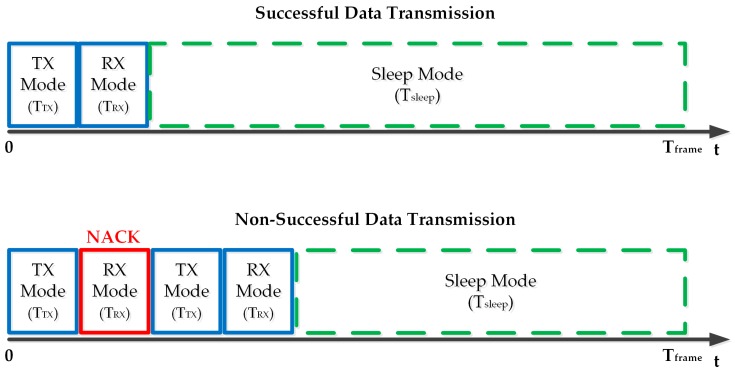
Illustration of the wrist-worn SpO2 RFIC transceiver activity in a successful data transmission case and in a non-successful data transmission case.

The wireless communications scenario considered is depicted in Figure 1, composed by two nodes, namely the wrist-worn wireless SpO2 sensor and the hub. For the application of interest, we assume that the wrist-worn wireless SpO2 sensor sends a data payload of 7 bytes in total with 3 data bytes for the measured SpO2 information and 4 bytes for the timestamp. The data payload is sent every $T_{\mathrm{frame}}\;$s. Once the Hub receives the data payload, it will reply with an acknowledgement (ACK) if the data packet is received with no errors, or else with a non-acknowledgement (NACK) if the received data packet contains errors. Figure 3 illustrates the RFIC transceiver activity in a successful and non-successful data transmission case. In particular, in a successful data transmission case, the wrist-worn wireless SpO2 sensor receives the ACK then it switches to sleep mode and it remains in this status until the new data packet transmission occurs. In a non-successful data transmission case, instead, the wrist-worn wireless SpO2 sensor receives a NACK packet, then it performs a retransmission of the data packet. 

In Table 4, a comparison among the protocols under evaluation in terms of calculated values for $T_{TX}\;$ and $T_{RX}$ as function of ACK Length, Data Packet Length and Data Rate is reported. The ACK length and Data Packet length values are derived from documents referenced in Protocol column of Table 4. The ACK Length is the number of bits of the ACK packet comprising of the overhead only. As for the Data Packet Length, it considers the sum of overhead plus payload bits. In the fifth column, $T_{RX}$ is obtained as the ratio of ACK Length to Data Rate. Similarly, in the sixth column $T_{TX}$ is obtained as the ratio of Data Packet Length to Data Rate. 

Finally, from the information in Table 4, the average RFIC current consumption $I_{avg}$ can be calculated as

(6) $$I_{\mathrm{avg}}=\;\frac{{(T}_{TX\;}I_{TX}{\;+\;T}_{RX}I_{RX}{)(1\;+PER)\;+\;T}_{\mathrm{sleep}\;}I_{\mathrm{sleep}\;}}{T_{\mathrm{frame}}}\;,$$

where

(7) $$T_{\mathrm{sleep}}{=\;T}_{\mathrm{frame}}\;-{\;(T}_{TX\;}{\;+\;T}_{RX})(1\;+\;PER).$$

For the calculation of $I_{\mathrm{avg}}$, the lowest possible data rate has been chosen while still ≥ 10 kbps [11]. A considerable difference in terms of Data Rate for ZigBee, BLE and WiFi can be noticed and this is due to the fact that the considered radio transceivers in Table 3 do not allow lowering the Data Rate below the values adopted in Table 4. On the other hand, using a larger Data Rate leads to a consistent reduction of δ, which determines a predominance of the contribution of the sleep current $I_{\mathrm{sleep}}$ with respect to the contribution of the transmitting current $I_{TX}$ and receiving current $I_{RX}$, and hence a lower $I_{\mathrm{avg}}$. As for $I_{TX}$, $I_{RX}$ and $I_{\mathrm{sleep}}$, the correspondent values reported in Table 3 have been used in the calculations that are now summarized.

**Figure 4:**
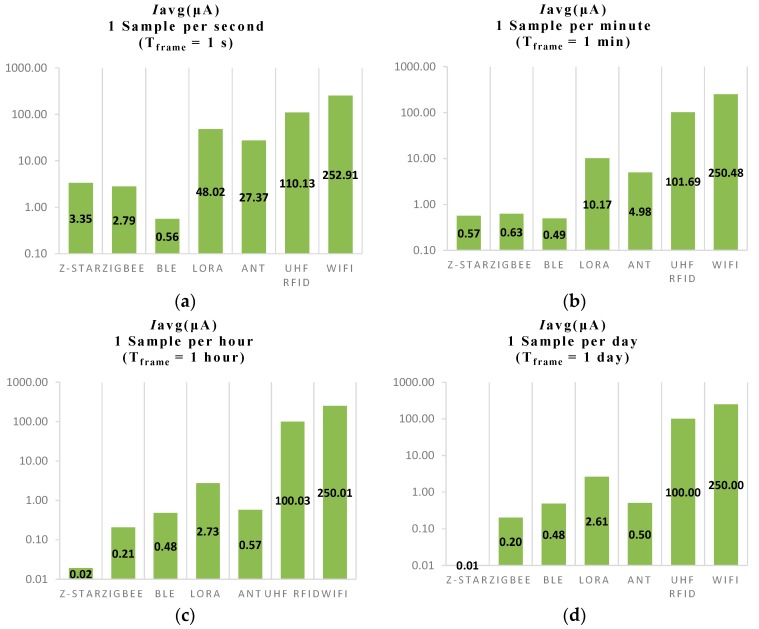
Histograms of the average current consumption: (**a**) $T_{\mathrm{frame}}\;=\;$1 s; (**b**) $T_{\mathrm{frame}}\;=\;$ 1 min; (**c**) $T_{\mathrm{frame}}\;=\;$ 1 h; (**d**) $T_{\mathrm{frame}}\;=\;$ 1 day.

Figure 4 shows four histogram graphs of $I_{\mathrm{avg}}$ for each protocol investigated considering four different $T_{\mathrm{frame}}$ values. In Figure 4a, a sampling rate of 1 sample per second with a $T_{\mathrm{frame}}$ of 1 s is considered for the calculation of $I_{\mathrm{avg}}$. In this particular case, it can be noticed that the radio transceiver that implements BLE protocol presents the lowest average current of 0.56 μA. Higher but still comparable values of $I_{\mathrm{avg}}$ are presented by the radio transceivers that implements ZigBee and Z-Star protocol that are respectively 2.79 μA and 3.35 μA. As for the ANT protocol, the corresponding radio transceiver has an $I_{\mathrm{avg}}$ of 27.37 μA, then the LoRa transceiver is characterized by an $I_{\mathrm{avg}}$ of 48.02 μA, the UHF RFID and WiFi transceiver by an $I_{\mathrm{avg}}$ greater than 100 μA. 

Figure 4b shows the values for each radio transceiver considering a sampling rate of 1 sample per minute or $T_{\mathrm{frame}}$ = 1 min. It can be noticed that in this case the situation among the $I_{\mathrm{avg}}$ values for the Z-Star, ZigBee and BLE radio transceiver changes considerably and are now within 140 nA of each other. As for LoRa and ANT, the correspondent $I_{avg}$ are significantly reduced but UHF RFID and WiFi are approximately the same as previously. In Figure 4c,d, a sample rate of 1 sample per hour and 1 sample per day have been considered. In these two final plots, it can be noticed that increasing $T_{\mathrm{frame}}$ for values greater than an hour lead to a significant reduction of duty cycle δ and hence the values of $I_{\mathrm{avg}}$ for each transceiver converge to correspondent value of$\;I_{\mathrm{sleep}}$. In conclusion, from the above results, a sampling rate of 1 sample per minute or greater is required to minimize the DC current consumption using the Z-Star Protocol. This sampling rate is suitable for a SpO2 measurement system as it represents a realistic interval between measurements. 

## 3. System Design

In this section, the design of a wrist-worn 915 MHz SpO2 sensor is presented. First of all, an overview of SpO2 will be given. The system architecture is then explained jointly with a description of the 3D system model used to design the 915 MHz antenna. Finally, the antenna topology will be described in detail. 

### 3.1. SpO2 Sensor Operation 

In this work, a wrist-worn reflectance-type pulse oximeter is proposed as an alternative to traditional transmittance-based pulse oximeters. A wrist-worn configuration is extremely desirable as it eliminates the requirement and discomfort associated with finger-mounted probes and allows for possibility of integration into a watch-type device. The additional SpO2 data allows blood oxygen parameters to be monitored during various physical activities that enables implementation of a wide range of new applications. These applications include monitoring of respiratory function, true rest and recovery times from strenuous sport activities, sleep apnea detection modes along with a wide range of medical conditions.

**Figure 5:**
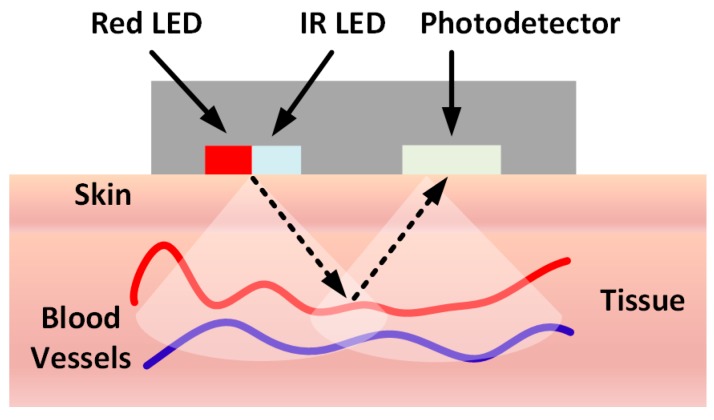
Working principle of SpO2 Sensor.

The PPG sensor monitors change in the light intensity via reflection from the tissue as illustrated in Figure 5. The SpO2 measurement is performed by illuminating the body surface (wrist in this case) with two wavelengths of light using Red and Infra-Red LEDs as shown. Reflected light from the skin, tissue and blood vessels is then captured using a photodetector [64]. The absorptive properties at each wavelength determine the level of oxygen saturation that is calculated from post-processing of measured signals. The signal amplitude is proportional to the quantity of blood flowing through the blood vessels. Each individual heartbeat is measured via the PPG sensor, and the signal is passed through digital filters to obtain the individual Systolic and Diastolic points for each wavelength. The PPG waveform has an alternating current (AC) component and a direct current (DC) component. The AC component corresponds to variations in blood volume synchronizations with the heartbeat. The DC component arises from the optical signals reflected by the tissues and is determined by the local optical scattering properties as well as venous and arterial blood volumes. Since multiple wavelengths are used, variations in skin tissue types and blood volumes are mostly canceled out due to Beer Lambert Modulation Ratios. A final calibration table relating to typical SpO2 Ratio between 660 nm and 940 nm allows a simple lookup table to be created showing the percentage of saturated blood oxygen with the measured patient. 

### 3.2. System Architecture and Electromagnetic Model

**Figure 6:**
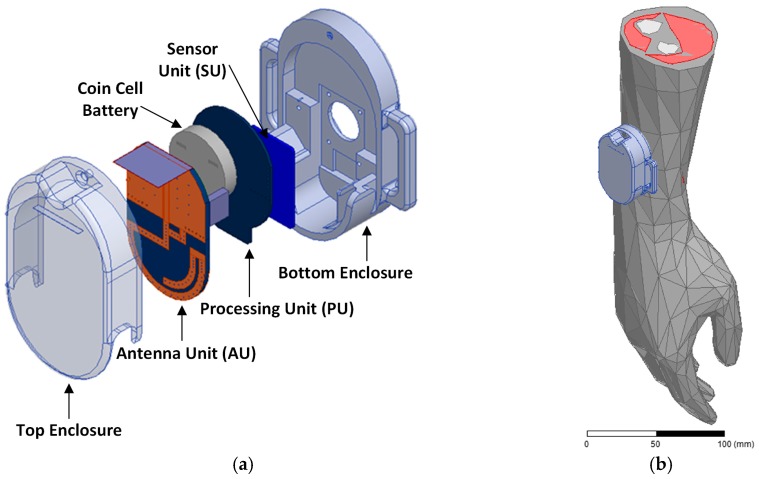
Wrist-worn wireless SpO2 sensor: (**a**) Exploded view of the Sensor Device; (**b**) Sensor device on voxel-based phantom arm.

In Figure 6, the wrist-worn wireless SpO2 sensor is depicted. From Figure 6a, which shows the exploded view of the sensor, the architecture of the whole system can be observed. In particular, it is characterized by three main units: Sensor Unit (SU), Processing Unit (PU) and Antenna Unit (AU). The SU is composed of the optical device placed in proximity of the wrist to measure the SpO2 level in the blood as explained above. The PU is placed on top of the sensor board and connected to it using a flex cable. This unit controls the SU and the information to be transmitted wirelessly. The AU allows wireless communication from the device either when it is worn on the wrist or in free-space. AU is interconnected to the PU by two standard 6-pin headers. The wrist-worn wireless SpO2 sensor is powered up by a coin cell battery placed between the PU and AU. The overall system is secured into a robust plastic enclosure and will be tied to the human wrist using straps.

Full-Wave Electromagnetic (EM) simulations of the system have been performed using ANSYS Electronics Desktop [65] in free space and on a phantom human wrist. To perform simulations in the latter case, a heterogeneous voxel-based model of the human arm, comprising bones, muscles, vessels, blood and skin has been used. In particular, in this case the system is placed on the forearm in proximity of the wrist as depicted in Figure 6b. The material used for the electromagnetic (EM) model for the enclosure of the wrist-worn wireless SpO2 sensor is Acrylonitrile Butadiene Styrene (ABS). The AU is implemented using standard low-cost FR4 substrate with copper metallization for radiator and ground plane. 

### 3.3. Antenna Topology

In this section, the design of an antenna integrated into the wrist-worn wireless SpO2 sensor device is discussed in detail. A challenge for the implementation of wireless communication in Sub-GHz bands in medical wearables is represented by the constraints on the antenna size. For instance, a short/medium range application at the MedRadio band or 433 MHz ISM band leads to a large antenna size. In fact, the free-space quarter-wavelength λ_0_/4 in these frequency bands varies from 16.4 to 18.7 cm. As for 915 MHz ISM band and 869.5 MHz Short Range Device band, $\;\lambda_{0}$/4 varies from 8.1 to 8.7 cm. Electrically small antennas [28] are therefore required at these frequencies for wrist-worn devices that are only several cm squared in size.

**Figure 7:**
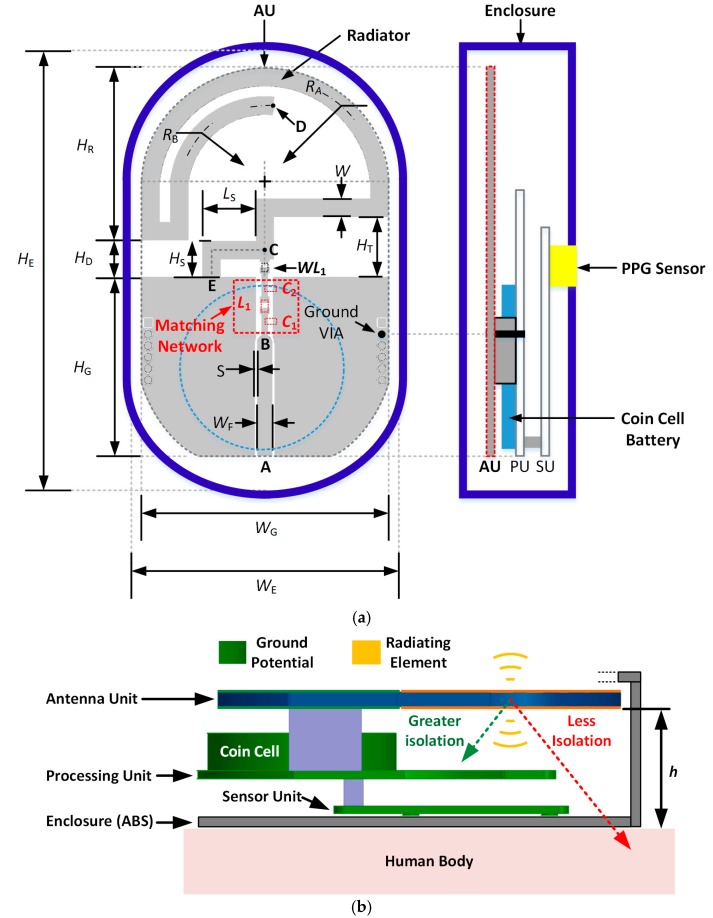
(**a**) Geometry of the proposed antenna: top view (**Left** side) and side view cross-section (**Right** side not to scale); (**b**) Improving antenna-body isolation.

The antenna is realized as a Printed Circuit Board (PCB) referred to as the AU and is shown with a red dashed outline in Figure 7a. The AU is placed on top of the PU and SU. All three functional units fit inside an enclosure of inside dimensions of *W_E_ × H_E_*. The antenna is realized using standard low-cost, FR-4 material with thickness *t* = 1.54 mm, ε_r_ = 4.17, tanδ = 0.016 and a copper thickness of 35 µm. The antenna feed is provided by an SMA connector located at Point A and connects to a 50 Ω grounded coplanar waveguide (GCPW) of width *W_F_* and spacing *S*. The GCPW then connects to the input of a π-type matching network shown at Point B. The output of the matching network drives the input of the radiator at Point C via a wire link *WL*_1_ that is an effective short-circuit at 915 MHz. Removal of *WL*_1_ enables direct measurement of the antenna input impedance at Point C using a Vector Network Analyzer (VNA). The chosen antenna topology was a Planar Inverted-F Antenna (PIFA), which is a variant of the quarter-wavelength monopole antenna, where the monopole is folded to be parallel to the ground plane and hence to reduce the height of the structure [66]. The chosen configuration presents a reduced radiated power in the direction of the ground plane as illustrated in Figure 7b which thus can be exploited as a partial-shield for the human body as in [67] as the PU, SU and coin-cell are placed between the radiator and body and are all at AC Ground potential. Isolation is also achieved by maintaining a physical separation (*h*) between the radiator and human body. The radiator element was designed using a curved inverted-F antenna (IFA) topology that follows the contour of the enclosure and achieves resonance close to the target frequency of 915 MHz. A shunt inductive microstrip branch is placed between point C and E similar to the classic IFA configuration to counteract the added capacitance that the curved radiator introduces. The top side radiator and ground plane geometries are mirrored on the bottom side with a series of 300 μm diameter plated-through via-holes used to connect both sides. The matching network was then designed in the circuit domain using Ansys Designer [68].

During Full-wave EM simulation, the resonant response of the antenna depends on two key parameters. The first is the total length of the radiating element denoted *l*_CD_. This length is defined as the total length of the radiating element, defined along the center line and can be seen from points C to D in Figure 7a. The second is the total length of the inductive branch from points C to E in Figure 7a and denoted *l*_CE_.

## 4. Simulation Results

In this section, simulated results in terms of |S_11_| of the proposed antenna are shown. Moreover, a method used to improve the impedance bandwidth characteristics of the antenna is explained. 

### 4.1. Return Loss

The following results show the simulated |S_11_| in free-space as a function of the key parameters of the antenna with all other parameters fixed as listed in Table 5. 

**Figure 8:**
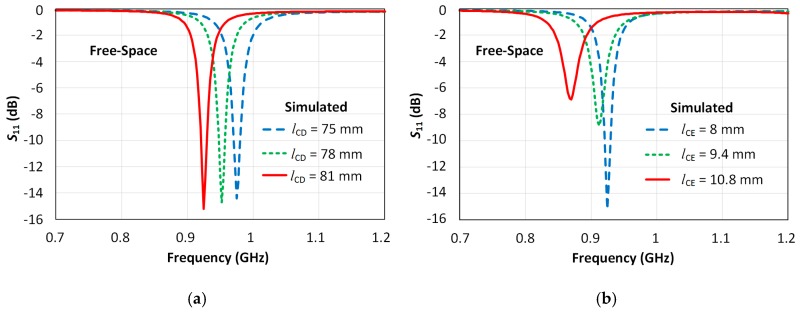

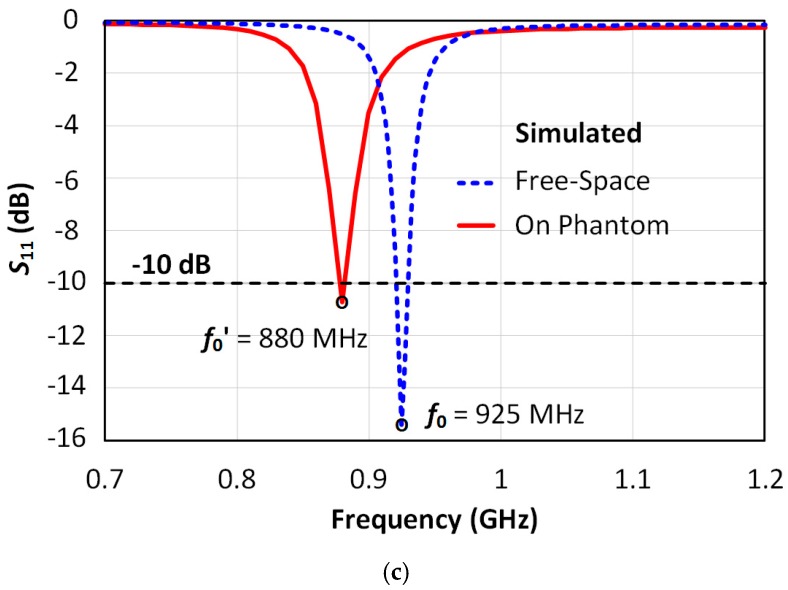
Simulated |S_11_|: (**a**) Simulated |S_11_| with parametric sweep of *l*_CD_ (*l*_CE_ fixed to 8 mm) in free- space; (**b**) Simulated |S_11_| with sweep of *l*_CE_ (*l*_CD_ fixed to 81 mm) in free-space; (**c**) Simulated |S_11_| with the final structure in free-space (dashed blue line) and on-wrist (solid red line).

Figure 8a shows the simulated |S_11_| as a function of the radiator length *l*_CD_, which was varied from 75 mm to 81 mm (considering *l*_CE_ fixed to 8 mm). The results show that increasing length *l*_CD_ leads to a reduction of the resonant frequency of the antenna. There is a small change in the minimum achievable |S_11_| value, showing that the impedance matching of the antenna is not greatly affected from *l*_CD_ with only a 1 dB variation observed in this case. Figure 8b shows a simulated parametric sweep as the length of the inductive branch *l*_CE_ was varied from 8 mm to 10.8 mm (considering *l*_CD_ fixed to 81 mm). The results show that this parameter *l*_CE_ plays a key role in determining both the resonant frequency and level of impedance matching that can be achieved. The simulations related to the results reported in Figure 8a,b were performed considering the system in free-space, moreover the sweeps are performed considering the parameter values reported in the third column of Table 5. 

The final values of parameters *l*_CD_ and *l*_CE_ were chosen to achieve a free-space resonant response close to 915 MHz with the final parameters shown in the right-most column of Table 5. The simulated response with optimized *l*_CD_ and *l*_CE_ values are shown in Figure 8c. In the free-space case (dashed blue line), it can be seen that the antenna is characterized by a −10 dB bandwidth of approximately 9 MHz which is less than the required value of 26 MHz at 915 MHz [12]. In addition, the on-wrist configuration shows that placing the antenna on the body results in a decrease in resonant frequency and impedance change of the antenna. These issues are addressed in the following section.

### 4.2. Impedance Bandwidth Improvement

**Figure 9:**
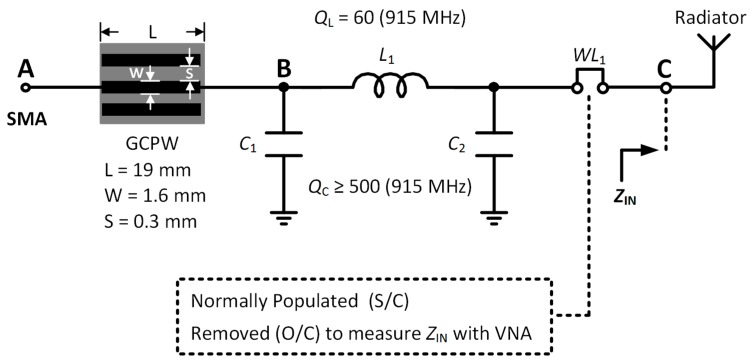
π-type matching network implemented in the sensor device.

In this section, a matching network is introduced to meet the impedance bandwidth requirement for the 915 MHz ISM Band. The main requirements for the matching network are that it provides the required impedance coverage with minimal loss over a specified bandwidth of at least 26 MHz from 902 to 928 MHz. Several matching network topologies are possible such as L-type, T-type, and π-type topologies. A low-pass, π-type matching network topology was chosen for this work as it has several desirable characteristics for this application. Firstly, it enables the Quality Factor (Q-factor) of the matching network to be specified by the user, unlike the L-type network. This in turn provides control of the impedance bandwidth. This topology also provides large impedance coverage, has good harmonic rejection capability, and has relatively small losses of a few tenths of a dB when high Q-factor microwave-grade components are selected [69]. The π-type matching network model is depicted in Figure 9 with the antenna feed denoted by SMA connector Port P_1_ at Point A. A short section of grounded coplanar waveguide (GCPW) connects the input to the matching network. The GCPW uses the same substrate as discussed previously relating to Figure 6. The matching network is composed of three reactive elements: a source-side shunt capacitor *C*_1_, a series inductor *L*_1_ and a load-side shunt capacitor *C*_2_. The quality (Q-factors) of the matching network components from American Technical Ceramics [56] model the losses of the components. Wire Link *WL*_1_ is short-circuited using a soldered connection by default and therefore has negligible effect on the matching network. Its purpose is to enable direct measurement of the antenna input impedance *Z*_IN_ when removed.

The function of the matching network is to match the antenna impedance *Z*_IN_ to the source impedance *Z*_S_ = 50 Ω of the radio front-end over a bandwidth greater than 26 MHz. The matching network was designed and optimized using ANSYS Designer [70]. The optimization consisted of minimizing the input reflection coefficient or *|S*_11_*|* over the ISM band from 902 to 928 MHz by varying the values of *C*_1_, *C_2_* and *L*_1_. The final values for the matching network components are listed in Table 6. 

## 5. Prototype Fabrication and Measurements

In this section, details on the wrist-worn wireless SpO2 sensor prototype fabrication and antenna characterization are presented. 

### 5.1. Prototype Fabrication

**Figure 10:**
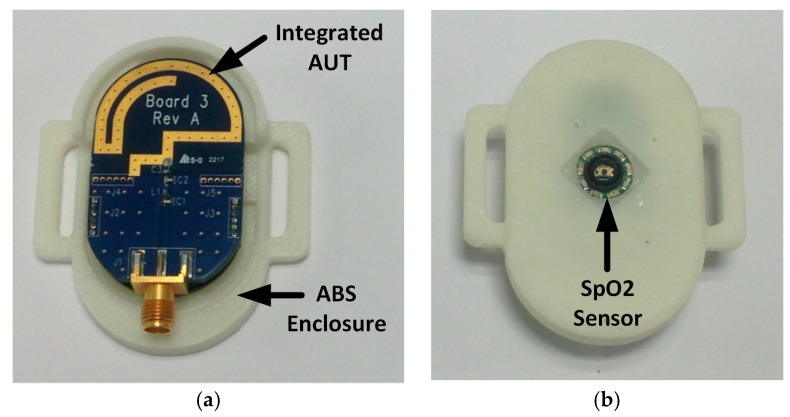

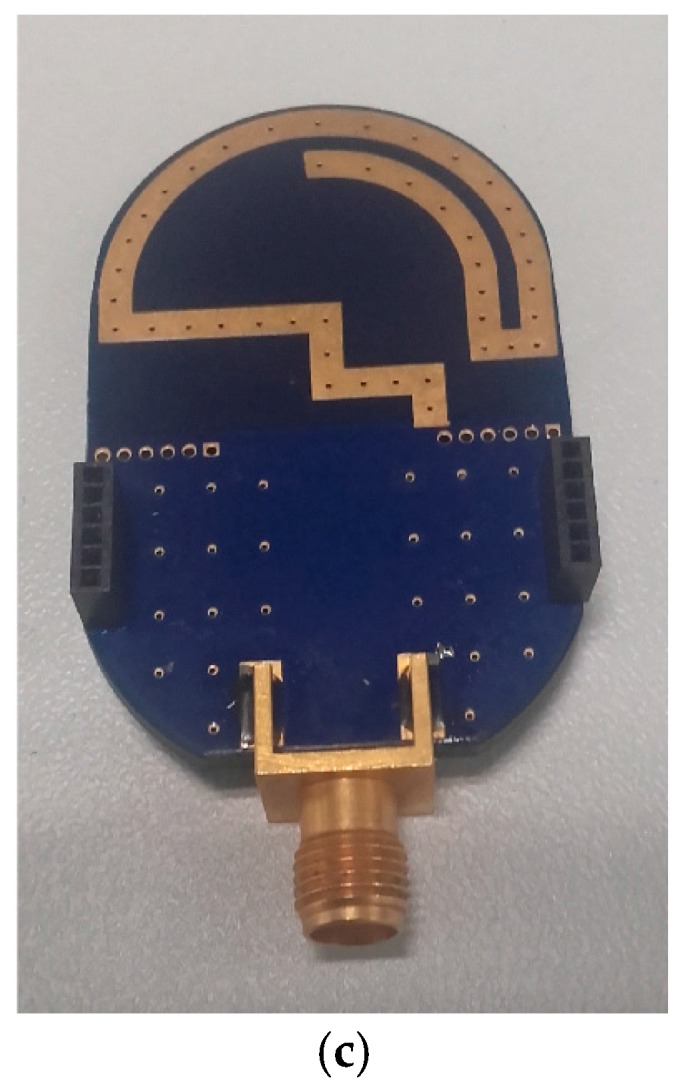
Fabricated wrist-worn wireless SpO2 sensor prototype: (**a**) top view with no top enclosure; (**b**) bottom view; (**c**) antenna PCB bottom view.

The top view and bottom view of the wrist-worn wireless SpO2 sensor with no top enclosure are shown respectively in Figure 10a,b. In Figure 10c the bottom view of the antenna PCB is depicted.

The integrated Antenna Under Test (AUT) has been fabricated on a 1.53 mm Isola 370-HR substrate [73]. Moreover, no soldermask has been deposited on the top of the radiator, where instead an Immersion Gold finish 3–7 µm is applied. Using this method, a reduction of the dielectric losses associated with the soldermask can be obtained. The matching network is placed close to the antenna feed point in the section highlighted also in Figure 7. The antenna is connected to the PU through two standard six-pin headers. The SpO2 sensor is placed underneath the processing board. The overall structure is secured to the enclosure using screws. The antenna is fed through a 50 Ω SMA connector for characterization purposes. From the bottom view, an aperture applied in the enclosure can be noticed. This aperture allows the SpO2 sensor measurements described in Section 3. 

### 5.2. Measured Impedance Characteristics

In this section, the measured antenna impedance characteristics *with* and *without* a matching network are presented. The measurements were conducted by placing the antenna within the SpO2 sensor and placing the assembly on a IXB-063 phantom column produced by INDEXSAR [74]. The IXB-063 phantom column comprises a silicon loaded with carbon powder and has been used to emulate the human wrist tissue effect to the antenna performance [74]. The IXB-063 phantom column is characterized by a cylindrical shape of 200 mm height and 63 mm diameter.

**Figure 11:**
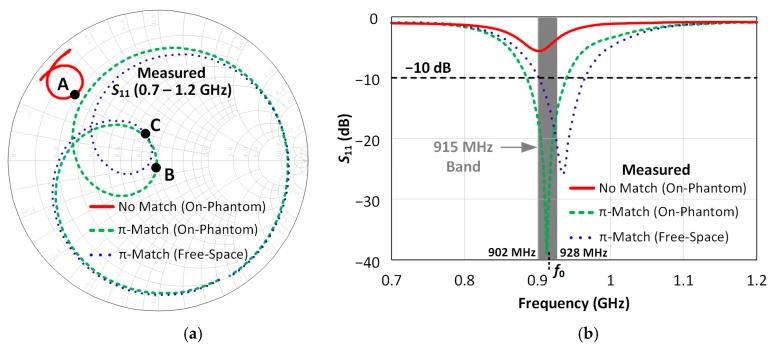
Impedance Matching: (**a**) Smith-Chart plot of the antenna impedance without matching network (solid red line), with the matching network on-phantom (dashed green line) and with the matching network in free-space (dotted blue line); (**b**) |S_11_| plot of the antenna impedance without matching network (solid red line), with the matching network on-phantom (dashed green line) and with the matching network in free-space (dotted blue line).

The Smith Chart of Figure 11a shows the measured antenna impedance on the phantom column with and without a matching network. With no matching network (solid red line), a large impedance mismatch can be observed at 915 MHz as shown at Point A. On the other hand, the addition of the π-type matching network (dashed green line) enables wideband matching around 915 MHz as shown at point B. In addition, the measured antenna impedance with the π-type matching network is shown (dotted blue) and shows only a small difference at 915 MHz (Point C) compared to on-body case, indicating a desirable resilience to detuning for the developed antenna.

The measured antenna |S_11_| is shown in Figure 11b both for the free-space and on IXB-063 phantom column cases. It can be observed that the antenna exhibits minimal detuning with the antenna maintaining a −10 dB impedance bandwidth of 55 MHz on the human arm and of 65 MHz in free-space. These figures meet the minimum 26 MHz bandwidth requirement for the 915 MHz band as defined in [12]. 

### 5.3. Radiation Pattern

A detailed explanation of the measurement setup with corresponding measurement results are presented in this section. 3D Radiation Patterns of the antenna system have been measured in an AMS-8050 Antenna Measurement System [75]. The measurements have been performed considering two different scenarios: in free-space and on IXB-063 phantom column. The radiation patterns are measured at a frequency of 915 MHz. Moreover, simulations of the radiation pattern for both cases in free-space and on an IXB-063 phantom column have been reported. For the simulation of the radiation pattern of the antenna on the IXB-063 phantom column in the 3D EM model the antenna system have been placed on a lossy cylinder with ε_r_ = 32.5 and σ = 0.5 S/m at 915 MHz, and same dimensions of the phantom according to the measurement setup. 

**Figure 12:**
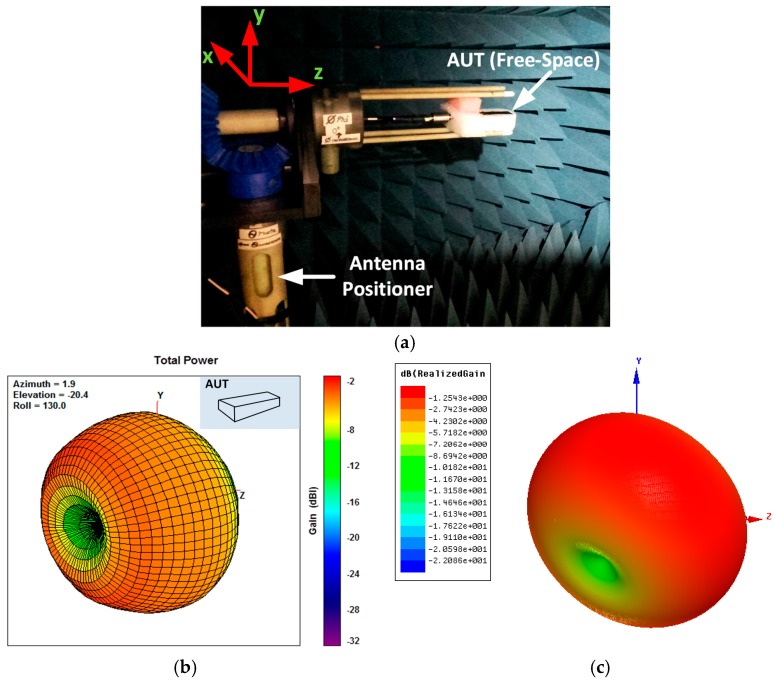
Radiation pattern measurements: (**a**) measurement setup in free-space scenario; (**b**) measured 3D radiation pattern of the antenna system in free-space; (**c**) simulated 3D radiation pattern of the antenna system in free-space.

In Figure 12a the 3D radiation pattern measurement setup in free-space scenario is depicted. The antenna system is positioned in order to rotate accordingly to the rotation of the multi-axis positioner (MAPS). The latter is characterized by a turret that enables rotation on the φ axis; the turret is integrated within a rotating table which enables rotation on the ϑ axis. The antenna system phase center is also aligned in order to make it be fixed with respect to the observation point during the measurements. In Figure 12b the measured 3D radiation pattern of the proposed antenna system in free-space is reported. The peak realized gain of the antenna results to be equal to −2.37 dBi at 915 MHz. It can be noticed also that the antenna is characterized by a dipole-like radiation pattern, with omnidirectional properties on the xy plane. In Figure 12c the simulated 3D radiation pattern of the proposed antenna system in free-space is reported. The simulated peak realized gain of the antenna results to be equal to −1.25 dBi at 915 MHz. The simulations show also that the antenna is characterized by a dipole-like radiation pattern, with omnidirectional properties on the xy plane. Slight differences can be noticed between measured and simulated radiation pattern due to the presence of the MAPS in the measurement system, which is not taken into account in 3D EM model used in simulation. 

In Table 7, a comparison between measured and simulated peak realized gain and directivity of the antenna in free-space is reported. A difference between measured and simulated peak realized gain of 1.12 dB and between measured and simulated directivity of 1.37 dB is observed. The difference in these values is mainly due to the fact that the losses associated with the SMA connector, the coaxial to GCPW transition and the matching network are not considered in 3D EM model.

**Figure 13:**
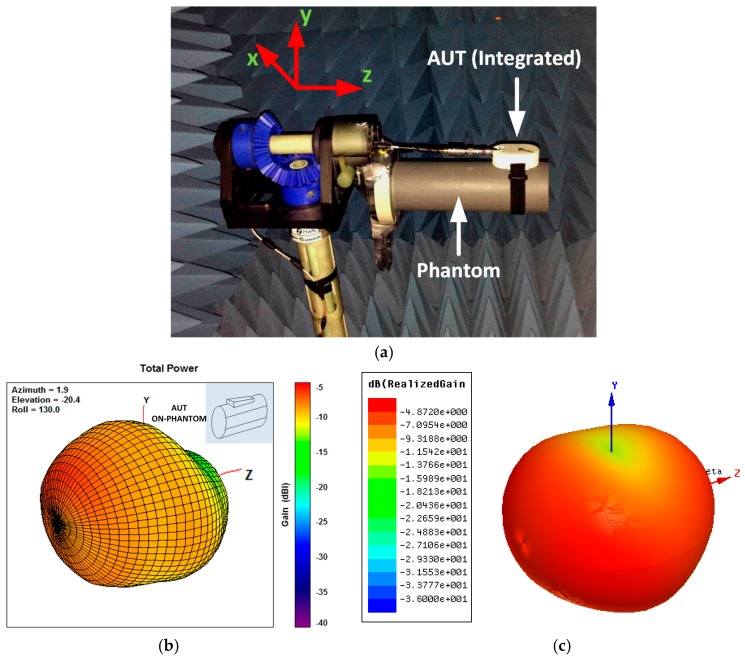
(**a**) antenna measurement setup on IXB-063 phantom column scenario; (**b**) measured 3D radiation pattern of the antenna system placed on IXB-063 phantom column; (**c**) simulated 3D radiation pattern of the antenna system placed on IXB-063 phantom column.

The 3D radiation pattern measurement setup of the antenna system on the phantom column IXB-063 scenario is depicted in Figure 13a. The test measurement setup is installed in such a way to make the phase center of the antenna be fixed with respect to the observation point during the measurements. The axis of the IXB-063 phantom column is aligned with the z-axis of the coordinate system used in the 3D Radiation pattern plots in Figure 13b. The antenna system is also secured to the IXB-063 phantom column using a wrist band strap. In Figure 13b the 3D radiation pattern of the antenna system on the IXB-063 phantom column is reported. The peak realized gain of the antenna in this case results to be equal to −6.1 dBi at 915 MHz. Therefore, there is a 3.6 dB decrease in gain because of the absorption due to the finite conductivity of the human wrist, which in this case is emulated by IXB-063 phantom column. Moreover, a distortion of the radiation pattern can be noticed which is due to the coupling of the radiator with the phantom column. It can be also noticed that the boresight direction is oriented to the rear-side of the antenna, which is due to the presence of the processing board as well as the ground plane and the coin cell battery that minimize the coupling between the antenna system and the phantom column. In Figure 13c the simulated 3D radiation pattern of the proposed antenna system on the phantom column IXB-063 scenario is reported. The simulated peak realized gain of the antenna results to be equal to −4.87 dBi at 915 MHz. The simulated radiation pattern presents similar characteristics compared to the measured one, with boresight direction oriented to the rear-side of the antenna. Also, in this case slight differences can be noticed between measured and simulated radiation pattern due to the presence of the MAPS in the measurement system, which is not taken into account in 3D EM model used in simulation. In Table 8, a comparison between measured and simulated peak realized gain and directivity of the antenna on the phantom column IXB-063 is reported. A difference between measured and simulated peak realized gain of 1.22 dB and between measured and simulated directivity of 1.38 dB is observed. Also in this case, the difference in these values is mainly due to the fact that the losses associated with the SMA connector, the coaxial to CPGW transition and the matching network are not considered in 3D EM model.

## 6. Discussion

To evaluate the potential of the 915 MHz ISM band with respect to the 2.45 GHz ISM band, in Section 2.1 a calculation of the link budget at 915 MHz and 2.45 GHz considering the theoretical gain defined by the size constraints of the wrist-worn wireless SpO2 sensor has been performed.

In this Section, the same calculation will be proposed but considering measured gain in free-space of the proposed antenna for the 915 MHz case compared with three antennas proposed in the literature, i.e., in [18,19,20], for the 2.45 GHz case. Moreover, the comparison is extended also to the on-body case. Note also that the antennas taken into account for comparison are characterized by a form factor slightly bigger than the proposed antenna. 

Specifically, in [18] Su and Hsieh demonstrate a 2.45 GHz loop antenna of dimensions 40 × 50 × 5 mm^3^ and Peak Gain of approximately 3.8 dBi at the operating frequency *f*_0_ in free space. In this work is reported also simulated Peak Realized Gain value of −0.89 dBi at *f*_0_ of the antenna on-body. In [19], Wu and Cheung present a cavity-backed annular slot antenna working at 2.45 GHz with dimensions ${\pi \;\times \;21}^{2\;}\times \;10$ mm^3^ and peak realized gain of approximately 3.5 dBi and 2.5 dBi at *f*_0_ for free-space and on-body scenario respectively. Finally, in [20], Wu, Wong et al., propose a 2.45 GHz internal shorted monopole antenna of dimensions 25 × 35 × 4 mm^3^ and peak realized gain of 1.5 dBi at *f*_0_. 

The scenario and conditions adopted in the calculation of Section 2.1 has been considered, namely a distance between transmitting antenna and receiving antenna *d* = 3 m, a receiving antenna characterized by a specified gain $G_{RX}$ of 0 dBi and a transmitted power $P_{TX}$ = 0 dBm.

A summary of the calculations performed using (3) is reported in Table 9. In particular, *G_TX−FS_* is the peak realized gain of transmitting antenna at *f*_0_ in free-space scenario, $G_{TX-OB}$ is the peak realized gain at *f*_0_ of the transmitting antenna on the body, $P_{RX-FS}$ is the calculated received power in the in free-space scenario and $P_{RX-OB}$ is the calculated received power in the on-body scenario. This comparison shows improved performance in terms of link budget at 915 MHz when compared with 2.45 GHz in the free-space. Specifically, the difference in received power ranges from 2.4 to 4.7 dB. As for the on-body scenario, the difference between the calculated received power in the 915 MHz case and in the 2.45 GHz case ranges from 0 to 3.4 dB. This shows that similar performance can be achieved at 915 MHz and 2.45 GHz under the assumption that the reader gain and received power remain the same.

## 7. Conclusions

In this paper, the potential of the Sub-GHz wireless communications is considered for future IoT wearable applications. This analysis has been performed to evaluate potential operational frequency bands for a wrist-worn wireless SpO2 sensor. The potential of a wrist-worn wireless SpO2 sensor for future IoT wearable medical applications has also been discussed. From the comparison between the two ISM bands under test, it has been shown that the 915 MHz ISM band has an 8.6 dB smaller free-space path loss when compared with 2.45 GHz. When the antenna gain limitation due to form factor restrictions is considered for the wrist-worn wireless SpO2 sensor, the 915 MHz ISM band offers a comparable link budget when compared to the 2.45 GHz ISM band. When evaluating various wireless protocols and radio transceivers, the results showed that average current consumption is a strong function of sleep current and sampling rate. The calculated results show that 2.45 GHz BLE protocol presents the lowest average current of 0.56 μA when sampling at 1 sample per second. On the other hand, when the sampling rate is reduced to 1 sample per minute, then Zigbee, BLE and Z-Star RFIC current consumption are approximately the same. Finally, when the sampling rate is reduced to 1 sample per hour or greater, the Sub-GHz Z-Star protocol leads to significantly less current consumption and this sampling rate is suitable for SpO2 measurement. This work also presents an antenna design for the wrist-worn wireless SpO2 sensor. Its dimensions are 44 × 28 × 1.6 mm^3^. It is designed to work both in free-space and on-human wrist scenarios in the 915 MHz ISM band. It presents a measured −10 dB impedance bandwidth of 55 MHz on the IXB-063 phantom column (used to mimic the effect of the human wrist on the antenna) and of 60 MHz in free space. The antenna is also characterized by a measured peak realized gain at 915 MHz of −6.1 dBi on IXB-063 phantom column and of −2.37 dBi in free space. This work also compares the developed antenna design with several other 2.45 GHz wrist-worn antennas from the literature. The results concluded that the designed 915 MHz antenna provides similar performance when compared with 2.45 GHz under the assumption that the reader gain and received power remain the same. The authors have also identified a number of challenges for the use of 915 MHz ISM band in a wrist-worn wireless SpO2 sensor. In fact, the gain figure and the −10 dB impedance bandwidth of an antenna at 915 MHz are limited by the form factor requirements. However a method to overcome the −10 dB impedance bandwidth have been proposed and implemented. Moreover, even though the gain figure of a 915 MHz is limited by the antenna dimensions, this frequency offers a lower free-space loss. 

In conclusion, the use of Sub-GHz frequencies for a wrist-worn SpO2 sensor is challenging due to physical size constraints that effects the antenna performance. However, the use of the 915 MHz Z-Star protocol shows that low power consumption can be achieved when compared with other wireless protocols under the constraint of requiring a maximum sampling rate of approximately 1 sample per minute. Future work regarding characterization of the final system implementation will be concerned with a detailed analysis of measured performance with respect to current consumption as well as a detailed investigation of human body effects on antenna performance. 

## Figures and Tables

**Table 1:** Link budget comparison for 915 MHz and 2.45 GHz ISM Band.

Parameter	Name (Units)	915 MHz ISM Band	2.45 GHz ISM Band	∆ (dB)
$P_{TX}$	Transmit Power (dBm)	0	0	0
FSPL	Free-Space Path Loss (dB)	41.22	49.78	8.6
$G_{TX}$ (1)	Calculated Tx Antenna Gain (dBi)	0.09	5.48	5.4
$G_{RX}$^1^	Rx Antenna Gain (dBi)	0	0, +3	0, +3
$P_{RX}$ (2)	Calculated Total Received Power (dBm)	−41.13	−44.3, −41.3	3.16, 0.16

^1^ Two values provided for *G*_RX_ (0 dBi, +3 dBi) at 2.45 GHz for comparison.

**Table 2:** Survey of potential Wireless Protocols for next generation Internet of Things.

Protocol	Operation Frequency Band (MHz)	Maximum Data Rate (kbps)	Typical Communication Range (m)	Topology	Ref.
Bluetooth BR/EDR	2400–2483.5	2100	10	P2P	[32]
BLE	2400–2483.5	2000	10	P2P; Star; Mesh	[32]
ZigBee	400–470; 800–960; 2400–2500	250	100	P2P; Star; Mesh	[14]
Z-Star	779–965	200	100	P2P; Star; Mesh	[33]
WiFi	2400–2500; 5725–5875	54$\;\times 10^{3}$	30	Star	[15,34]
HaLow	755–928	8670	100	Star	[35]
ANT/ANT+	2400–2457	60	5	P2P; Star; Mesh	[36]
LoRa	135–175, 410–525, 779–787, 863–869, 902–928	300	2000–5000	Star	[37,38]
UHF RFID	860–960 MHz	640	15	Star	[39,40]

**Table 3:** Survey of low-power RF Integrated Circuits transceivers available commercially.

Wireless Protocol	Output Power (dBm)	Typical Current Consumption in RX Mode (mA)	Typical Current Consumption in TX Mode ^1^ (mA)	Typical Current Consumption in Sleep-Mode (μA)	RFIC Ref
BLE	0	3.7	3.4	0.48	[45]
ZigBee	−28 to +4.5	27	28	0.2	[46]
Z-star	−25 to 0	2.4	5.3	0.01	[33]
WiFi ^2^	0 to +10	115	115	250	[47]
ANT/ANT+ ^2^	−6 to +4	23.7	28.8	0.5	[48]
LoRa	−4 to +14	9.9	21.2	2.6	[38,49]
UHF RFID	−6 to +12	18	18	100	[50]

^1^ The current consumption is related to a transmission power of 0 dBm except for the ZigBee and LoRa transceiver for which it is related to a transmitted power of −0.5 dBm and −0.6 dBm respectively. ^2^ The device considered is a full transceiver module.

**Table 4:** Comparison of $T_{TX}\;$and $T_{RX}$ as function of ACK Length, Data Packet Length and Data Rate.

Protocol	ACK Length (bits)	Data Packet Length (bits)	Data Rate (kbps)	$T_{\mathrm{RX}}$ (ms)	$T_{\mathrm{TX}}$ (ms)
Z-Star [33]	176	232	50	3.52	4.64
ZigBee [56,57]	88	144	250	0.352	0.576
BLE [56,58]	80	160	1000	0.08	0.160
LoRa [38,59]	120	176	10.9	11	16.1
ANT [48,60]	48	88	13.8	3.48	6.38
UHF RFID [40,47,61,62]	22	88	19.2	1.15	4.58
WiFi [47,63]	112	168	1000	0.112	0.168

**Table 5:** Tabulated values of the antenna physical parameters (free-space).

Parameter Name	Swept Parameter	Starting Value (mm)	Final Optimized Value (mm)
W_G_	N	27.4	27.4
W_E_	N	37	37
W_F_	N	1.6	1.6
W	N	2	2
H_E_	N	59	59
H_R_	N	19.1	19.1
H_G_	N	19.4	19.4
H_T_	N	6	6
Ls	Y	6–7.4	6
Hs	Y	4–5.4	4
R_A_	N	11.9	11.9
R_B_	N	8.5	8.5
H_D_	Y	4–7	4
*l*_CD_	Y	75–81	81
*l*_CE_	Y	8–10.8	8

**Table 6:** List of the component values used in the matching network.

Component Label	ATC Part Number	Value
*C*_1_	ATC 600L 1R5BT [71]	1.5 pF
*C*_2_	ATC 600L 5R6BT [71]	5.6 pF
*L*_1_	ATC 0603WL8R2JT [72]	8.2 nH

**Table 7:** Comparison between measured and simulated radiation characteristics of the antenna in free-space.

	Measured	Simulated	∆ (dB)
Peak Realized Gain (dBi)	−1.25	−2.37	1.12
Directivity (dBi)	1.78	3.15	1.37

**Table 8:** Comparison between measured and simulated radiation characteristics of the antenna on the IXB-063 phantom column.

	Measured	Simulated	∆ (dB)
Peak Realized Gain (dBi)	−4.87	−6.09	1.22
Directivity (dBi)	2.33	3.71	1.38

**Table 9:** Summary of the link budget calculation considering the proposed antenna and antennas demonstrated in the literature.

Ref.	Antenna Description	Dimensions (mm^3^)	$f_{0}$ (MHz)	$G_{TX-FS}$ (dBi)	$G_{TX-OB}$ (dBi)	$P_{RX-FS}$ (dBm)	$P_{RX-OB}$ (dBm)
[18]	Loop	40 × 50 × 5	2450	~+3.8	−0.89 ^1^	−46	−50.7
[19]	Cavity-backed annular slot	${\pi \;\times \;21}^{2\;}\times \;10$	2450	~+3.5	~+2.5	−46.3	−47.3
[20]	Internal shorted monopole	25 × 35 × 4	2450	+1.5	−	−48.3	−
Proposed	Curved Inverted-F	44 × 28 × 1.6	915	−2.37	-6.1	−43.6	−47.3

^1^ Simulated value.
